# Supplementary material for: Lupane Triterpene Derivatives Improve Antiproliferative Effect on Leukemia Cells through Apoptosis Induction
Source: Molecules. 2022 Nov 26;27(23):8263. doi: 10.3390/molecules27238263 (PMC9738192; doi:10.3390/molecules27238263)
Supplement: Supplementary file 1 [file molecules-27-08263-s001.zip › molecules-2041466-supplementary.pdf]

Valencia-Chan et al. Supporting information

# Mechanism of action on leukemia cell lines of some derivatives of lupane triterpenes isolated from *Phoradendron wattii*

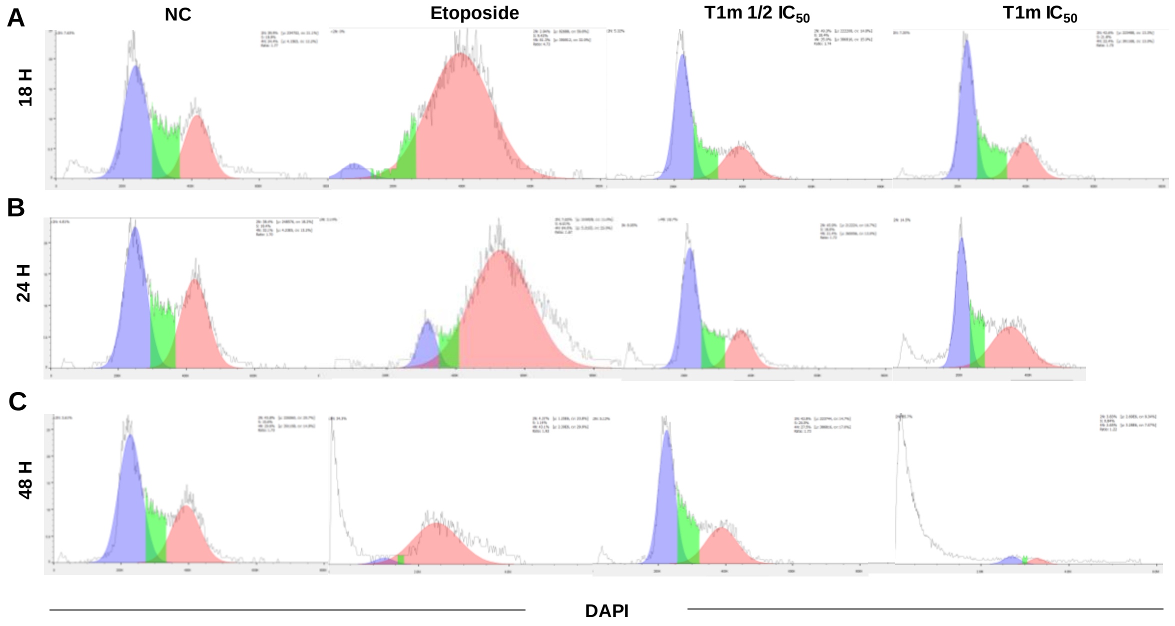


**Figure S1.** Effect of **T1m** on the cell cycle at two different concentrations and different times of culture (A: 18 h, B: 24 h and C: 48 h). Cell cycle status was analyzed by DAPI staining for three replicates, figure show a representative histograms.

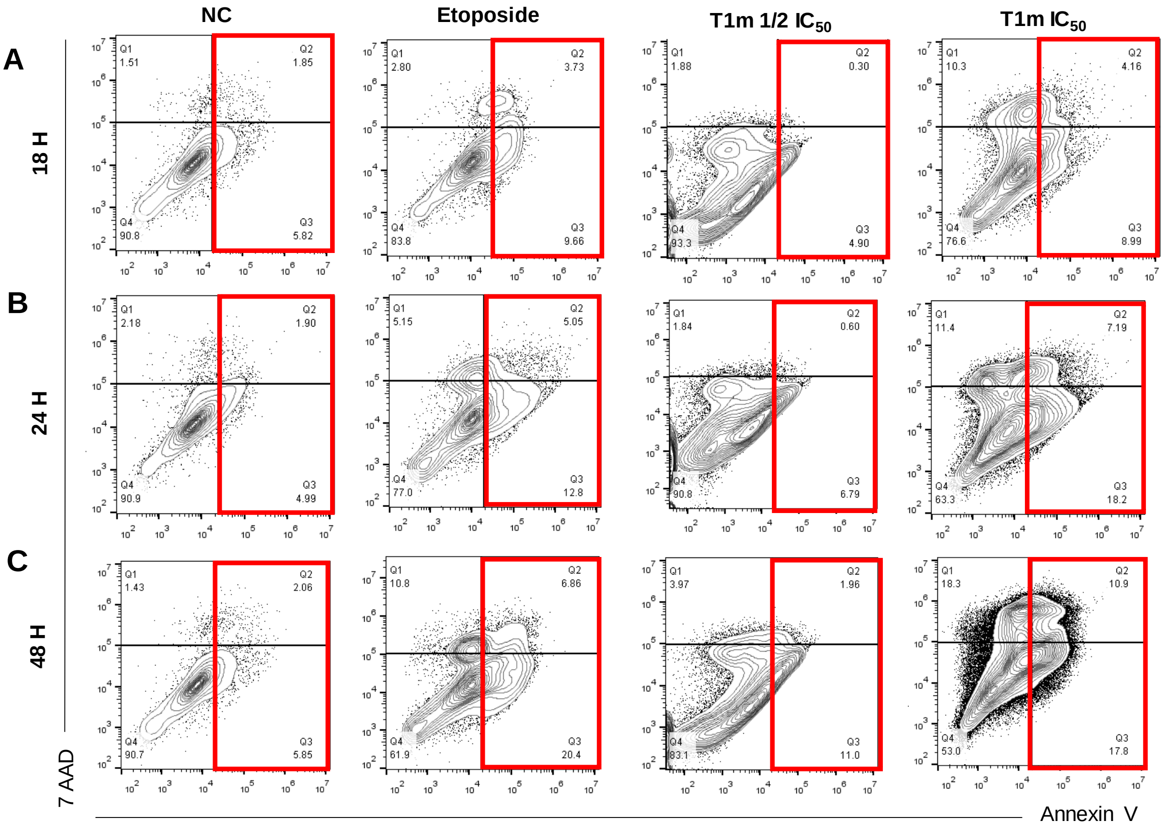


**Figure S2.** Effect of **T1m** in cell death apoptosis. K562 cell lines were cultured 18 (A), 24 (B) and 48 h (C) in presence of different concentrations of **T1m**. Apoptotic cell population in red box (positive annexin V-FITC) and necrotic population (single positive 7-AAD).

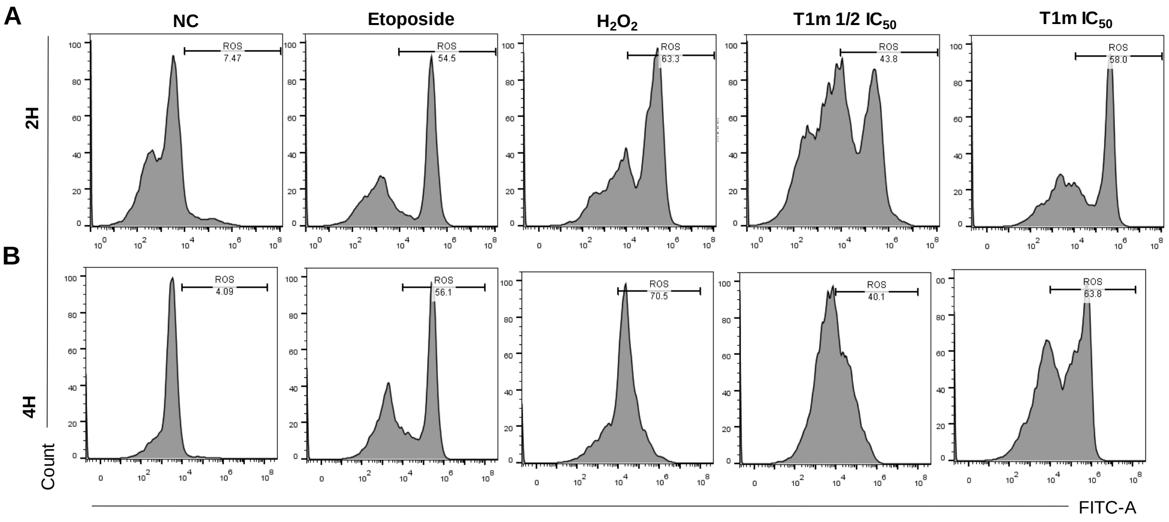


**Figure S3.** Effect of **T1m** on the generation of ROS at two concentrations and two times exposure (A: 2h and B: 4h), using CM-H2DCFDA. The histograms presented correspond to the representation of three different replicas.

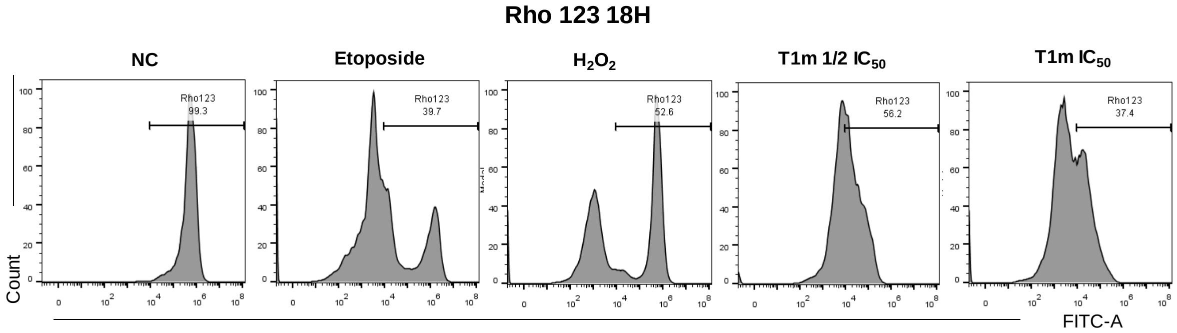


**Figure S4.** Effect of **T1m** on loss of mitochondrial membrane potential (ΔψM) at two different concentrations and two times of 2h (A) and 4h (B) of exposure, using Rhodamine 123. The histograms presented correspond to the representation of three different replicas.

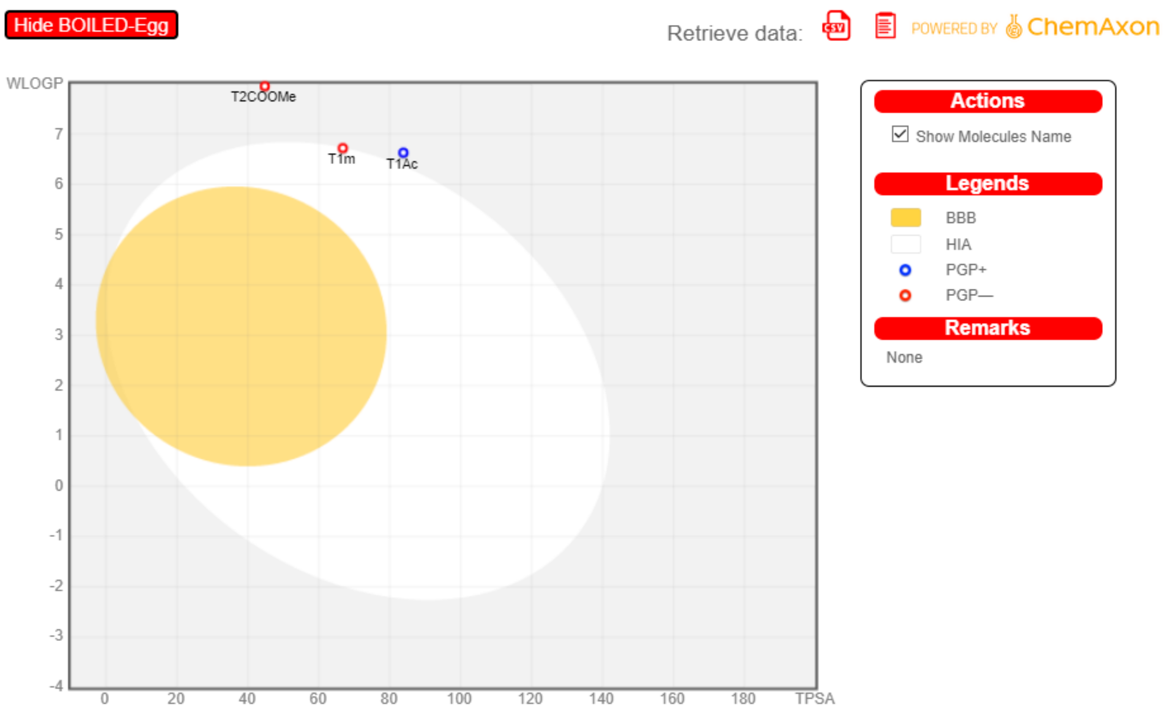


**Figure S5.** Boiled-egg graph from SwissADME prediction for triterpene derivatives. BBB: blood brain barrier, HIA: gastrointestinal absorption, PGP+: substrate of P-glycoprotein, PGP -: non-substrate of P-glycoprotein.

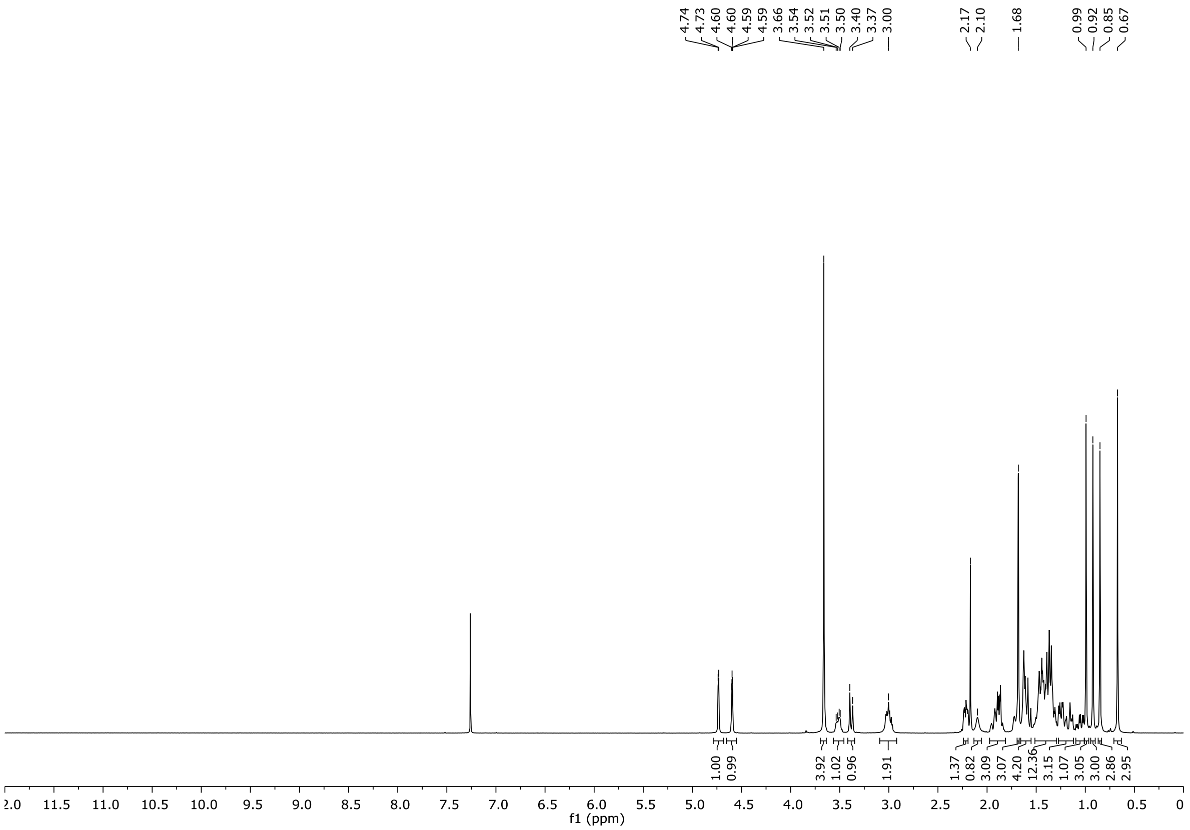

**Figure S6.** $^{1}$H-NMR (400 MHz, $CDCl_3$) spectrum of 3α-metoxy-24-hydroxylup-20(29)-en-28-oic acid (**T1m**).

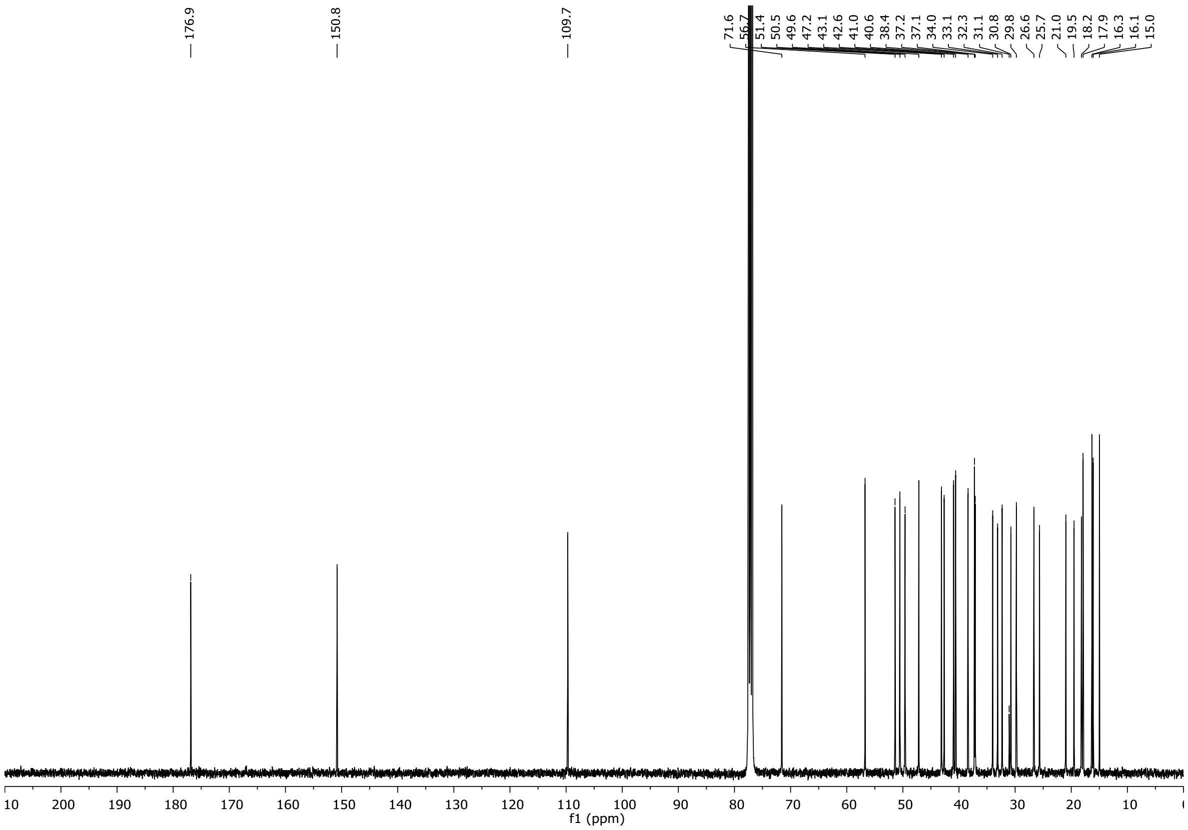


**Figure S7.** $^{13}$C-NMR (100 MHz, $CDCl_3$) spectrum of 3α-metoxy-24-hydroxylup-20(29)-en-28-oic acid (**T1m**).

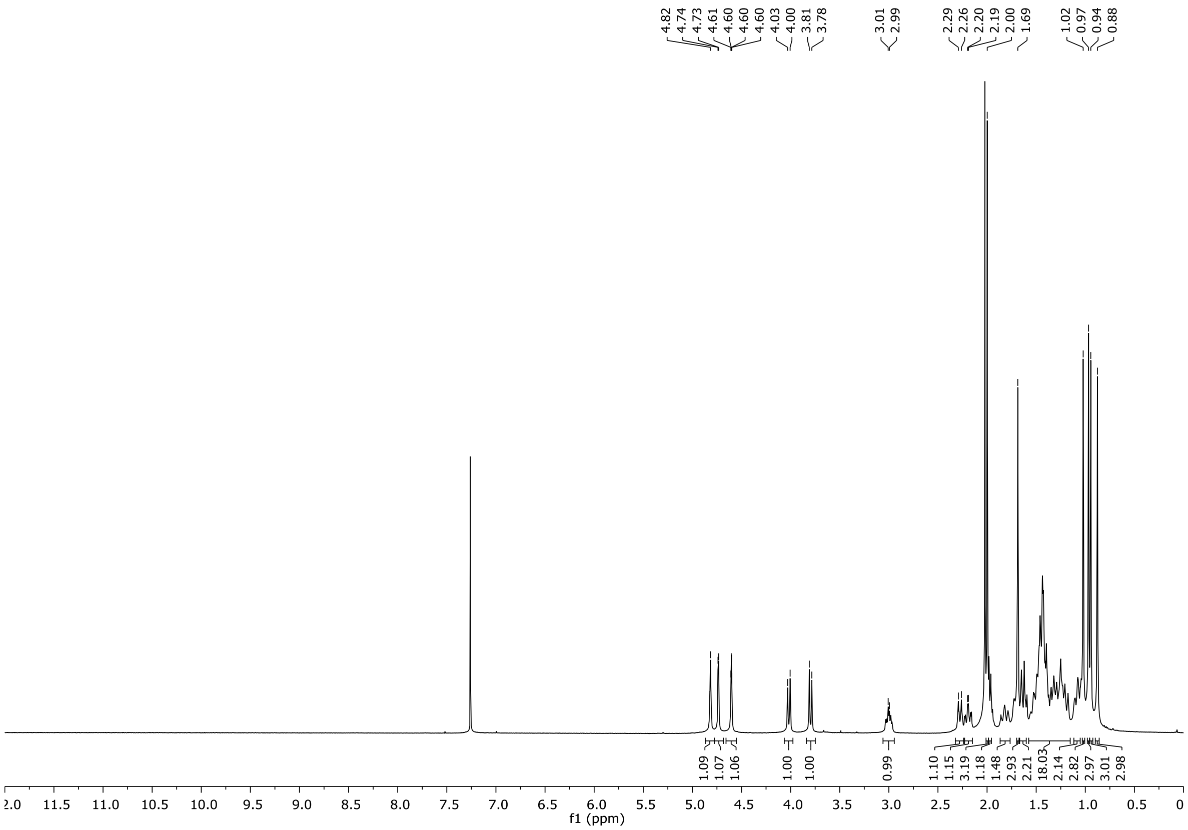


**Figure S8.** $^{1}$H-NMR (400 MHz, $CDCl_3$) spectrum of 3α-acetyl-24-hydroxylup-20(29)-en-28-oic acid (**T1Ac**).

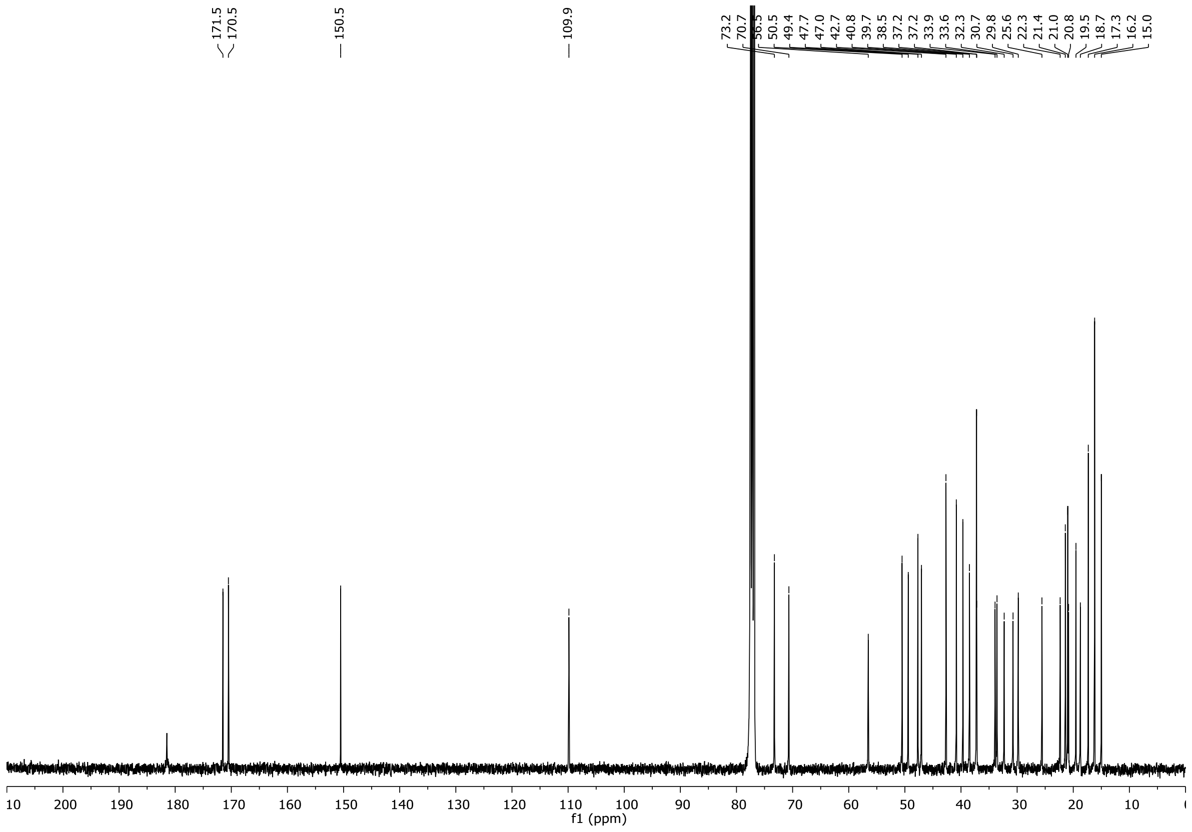


**Figure S9.** $^{13}$C-NMR (100 MHz, $CDCl_3$) spectrum of 3α-acetyl-24-hydroxylup-20(29)-en-28-oic acid (**T1Ac**).

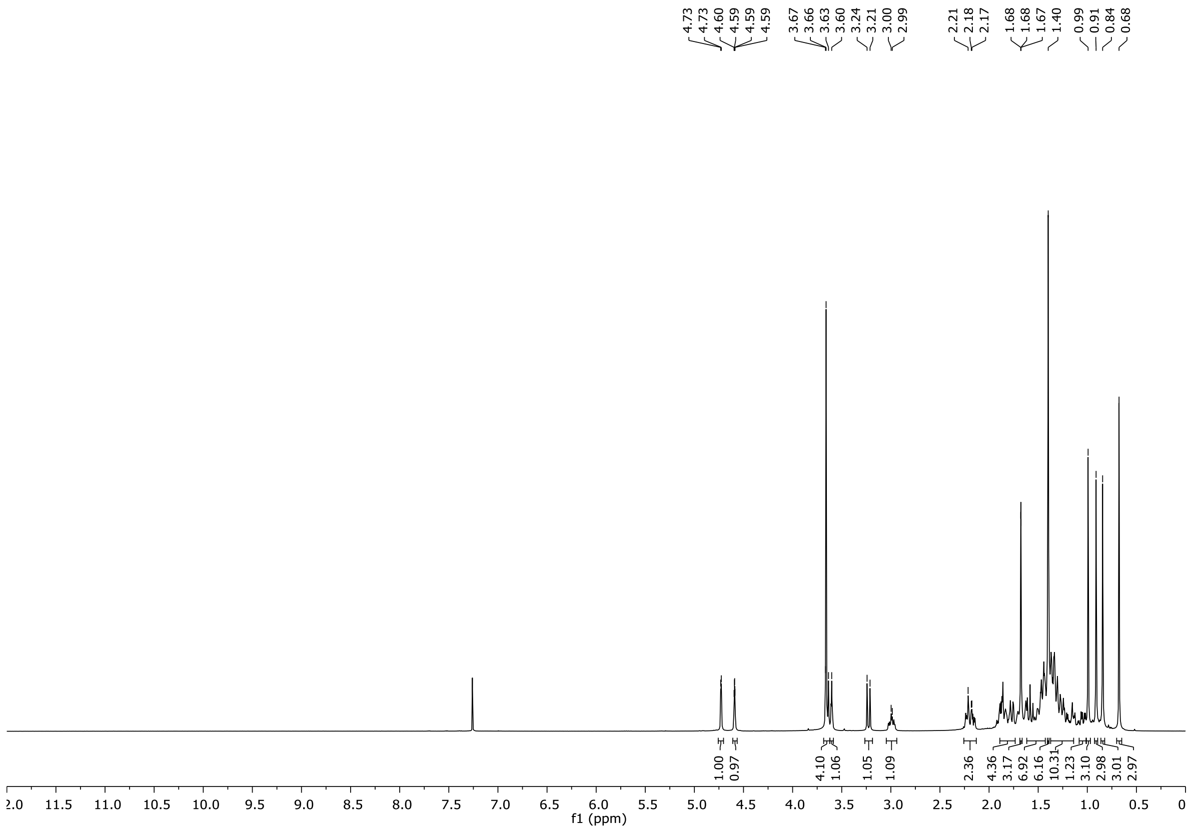


**Figure S10.** $^{1}$H-NMR (400 MHz, $CDCl_3$) spectrum of metyl ester of 3α,23-*O*-isopropylidenyl-3α,23-dihydroxylup-20(29)-en-28-oic acid (**T2m**).

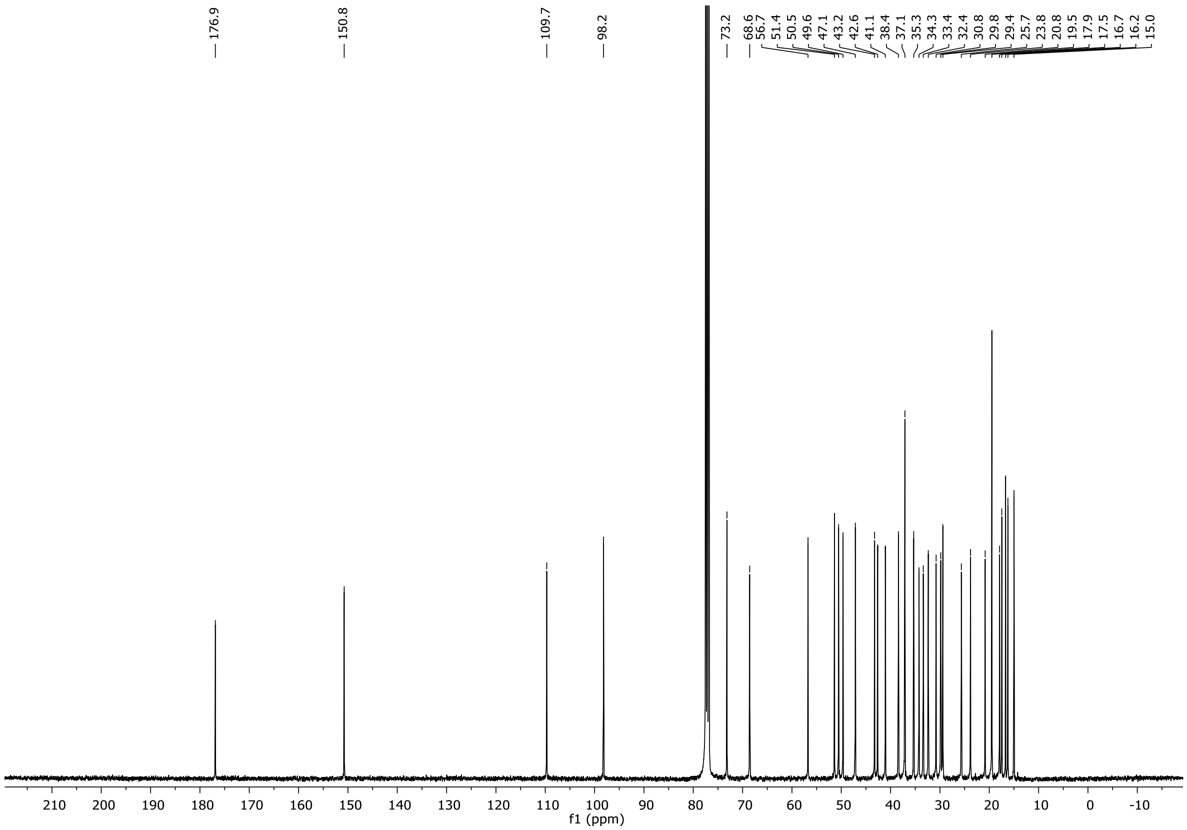


**Figure S11.** $^{13}$C-NMR (100 MHz, $CDCl_3$) spectrum of metyl ester of 3α,23-*O*-isopropylidenyl-3α,23-dihydroxylup-20(29)-en-28-oic acid (**T2m**).
